# A new brood-pollination mutualism between *Stellera chamaejasme* and flower thrips *Frankliniella intonsa*

**DOI:** 10.1186/s12870-021-03319-5

**Published:** 2021-11-29

**Authors:** Bo Zhang, Shu-Fan Sun, Wang-Long Luo, Jia-Xin Li, Qiang-En Fang, De-Gang Zhang, Gui-Xin Hu

**Affiliations:** Key Laboratory of Grassland Ecosystem of Ministry of Education, and Sino-U.S. Centers for Grazingland Ecosystem Sustainability, College of Grassland Science, Gansu Agricultural University, Lanzhou, 730070 China

**Keywords:** *Stellera*, Mutualism, Plant-pollinator interaction, Pollination, Reproductive fitness, Thrips

## Abstract

**Background:**

Brood pollination mutualism is a special type of plant-pollinator interaction in which adult insects pollinate plants, and the plants provide breeding sites for the insects as a reward. To manifest such a mutualism between *Stellera chamaejasme* and flower thrips of *Frankliniella intonsa*, the study tested the mutualistic association of the thrips life cycle with the plant flowering phenology and determined the pollination effectiveness of adult thrips and their relative contribution to the host’s fitness by experimental pollinator manipulation.

**Results:**

The adult thrips of *F. intonsa*, along with some long-tongue Lepidoptera, could serve as efficient pollinators of the host *S. chamaejasme*. The thrips preferentially foraged half-flowering inflorescences of the plants and oviposited in floral tubes. The floral longevity was 11.8 ± 0.55 (mean ± se) days, which might precisely accommodate the thrips life cycle from spawning to prepupation. The exclusion of adult thrips from foraging flowers led to a significant decrease in the fitness (i.e., seed set) of host plants, with a corresponding reduction in thrips fecundity (i.e., larva no.) in the flowers.

**Conclusions:**

The thrips of *F. intonsa* and the host *S. chamaejasme* mutualistically interact to contribute to each other’s fitness such that the thrips pollinate host plants and, as a reward, the plants provide the insects with brooding sites and food, indicating the coevolution of the thrips life cycle and the reproductive traits (e.g., floral longevity and morphology) of *S. chamaejasme*.

**Supplementary Information:**

The online version contains supplementary material available at 10.1186/s12870-021-03319-5.

## Background

Brood pollination mutualism is a special type of plant-pollinator interaction in which adult insects pollinate plants, and the plants provide breeding sites for the insects as a reward [1]. These mutualistic interactions are regarded as influential study systems for coevolutionary biology, fascinating natural historians, ecologists, and evolutionary biologists. The high diversity of mutualisms can provide opportunities to understand the mechanisms by which both plants and insects diversify and the role of biotic interactions in diversification.

In recent decades, a few brood-pollination mutualisms have been documented [reviewed by 1]. These mutualisms can be classified into two groups according to the food that host plants provide for the larvae of pollinators [see [2]]. In the first group, the pollinators rear their offspring on seeds (or ovules) of the host plants. The best-known examples are mutualisms between fig trees (*Ficus*) and fig wasps [3] or between yuccas (*Yucca*) and yucca moths [4–6], both of which are thought to be the product of coevolution [7]. Such mutualisms also occur between saxifrages (*Saxifraga*) and *Greya* moths [8], globeflowers (*Trollius*) and globeflower flies [9–11], Senita cacti (*Lophocereus*) and senita moths [12], leaf-flower plants (*Phyllanthus*) and leaf-flower moths [13, 14], and *Silene* and two genera of moths, *Hadena* and *Perizoma* [15]. In the second group, the larvae of pollinators feed on the pollen grains of host flowers, and these insects are almost all thrips (Thysanoptera) [2]. Compared to the first group, plant-thrips mutualisms have received less attention, as thrips were long believed not to serve as efficient pollinators due to their tiny body size and limited ability to move. To date, although the role of thrips in plant pollination has been documented in a few studies [16–18], detailed research on mutualistic interactions between plants and thrips is lacking [but see [19, 20]], and the interactive effect on trait evolution of both partners is little known. In this study, we explored and presented a new case of brood-pollination mutualism occurring between *Stellera chamaejasme* and *Frankliniella intonsa* (thrips).

*S*. *chamaejasme*, the only species of the genus *Stellera *in the mainland of China, is a multistemmed perennial herb. The plant is self-incompatible, and its reproduction completely relies on seeds, i.e., sexual reproduction. Traditionally, the species was believed to be pollinator-specialized, i.e., pollinated by a long-tongued lepidopteran (moths and butterflies) owing to its long-tubed flowers [21]. However, the plant also exhibits thrips pollination syndrome in view of its enclosed floral morphology with a narrow entrance that may provide shelter for the eggs and larvae of thrips, and other relevant traits, such as white to yellow sweet-scented flowers [see [17, 22]]. In addition, it has been commonly observed in natural populations that many thrips were present in inflorescences of *S*. *chamaejasme*, with their larvae inhabiting floral tubes, and that the plants still had a high reproductive fitness (i.e., seed set), even though few butterflies or moths were present in a population. Based on these observations, we hypothesized that *S. chamaejasme* and thrips mutualistically interacted and contributed to each other’s reproductive success. In the present study, we determined the contribution of thrips to the reproductive fitness of *S*. *chamaejasme* by pollination manipulation and identified their mutualistic relationship by associating the life cycle of the thrips with the flowering phenology of the plant. Specifically, we addressed three questions: 1) Are thrips efficient pollinators for *S*. *chamaejasme*? 2) Does there exist mutualistic interaction, i.e., brood-pollination mutualism between the plant and *F. intonsa*? 3) If yes, how is life cycle of the thrips related to the flowering phenology of *S. chamaejasme*?

## Results

### Flowering and pollination of *S. chamaejasme*

In the population studied, *S. chamaejasme* flowered from late June (June 20) to the end of July. For individual inflorescences, the flowering period from the first flower opening to the last flower wilting lasted 21.3 ± 0.24 (mean ± SE, *n* = 50) days. It took 2.3 ± 0.11 days from anthesis of the first flower to the half-flowering stage, 10.5 ± 0.31 days to the full-flowering stage, and 10.8 ± 0.27 days from the full-flowering stage to the wilting stage. For a single flower, its longevity was 11.8 ± 0.55 (mean ± SE, *n* = 9) days.

**Fig. 1:**
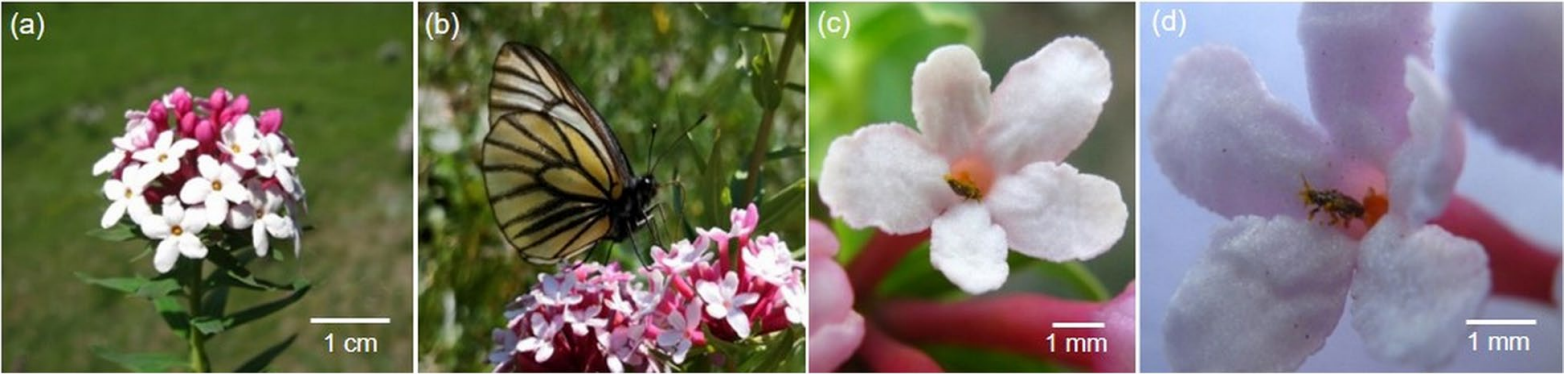
Floral characteristics and pollination by different types of pollinators in *S. chamaejasme*. **a** An inflorescence; (**b**) pollination by a butterfly (*Aporia crataegi*); (**c**, **d**) a single flower, pollinated by thrips *F. intonsa*

There were two types of pollinators observed in the studied population of *S. chamaejasme* (Fig. 1). One type was butterflies (Fig. 1b), and the other was thrips of *F. intonsa* (Figs. 1c, d). The butterflies included three species, i.e., *Aporia crataegi*, *Aglais urticae* and *Vanessa cardui*, which accounted for 46, 40 and 14% of the total visits of lepidopteran insects, respectively. Thrips could be observed in fresh flowers of the half-flowering inflorescences, carrying much pollen on their bodies when climbing out of floral tubes.

### Life-history activities of *F. intonsa* at different flowering phases of *S. chamaejasme*

**Fig. 2:**
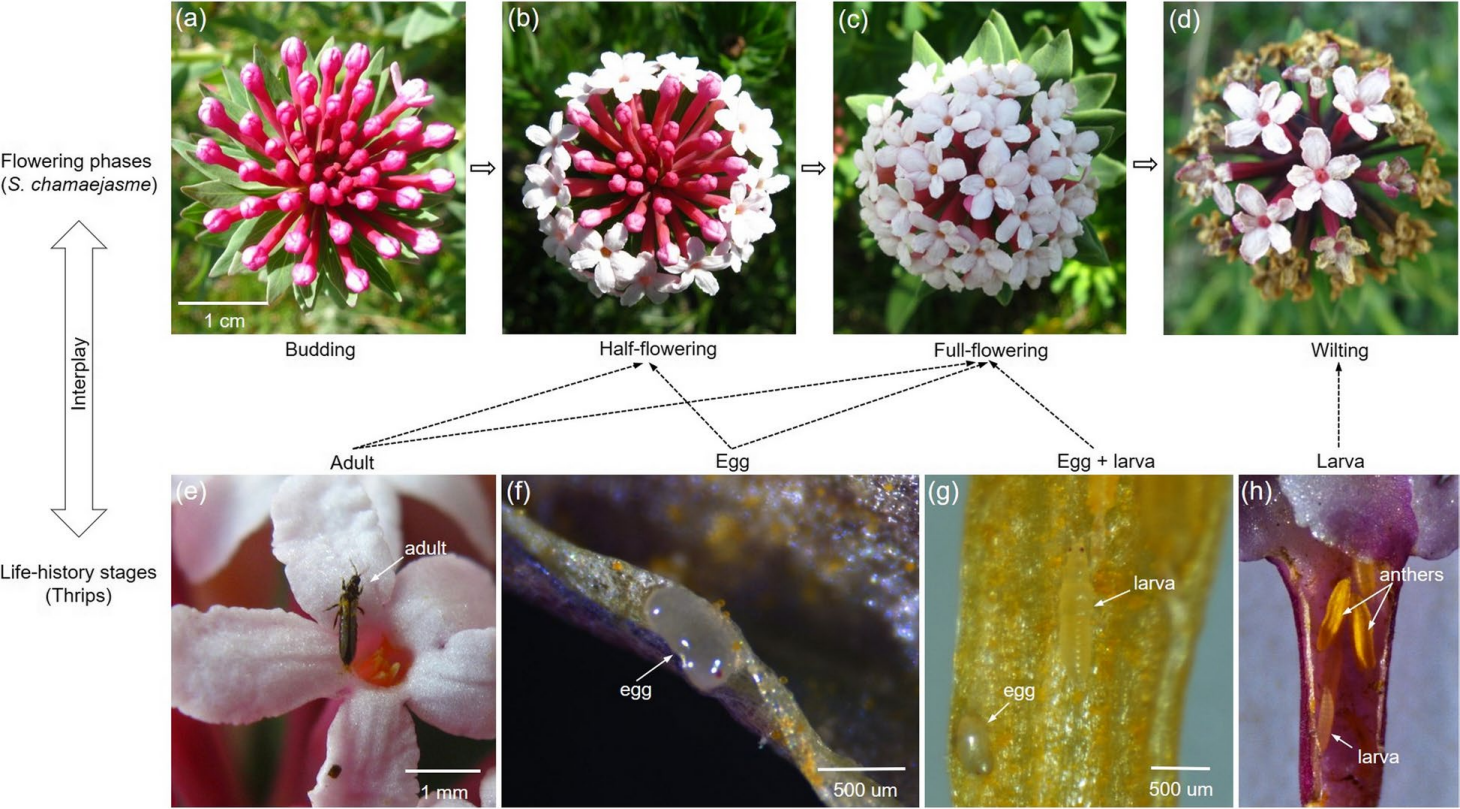
Mutualistic association of different flowering phases of an inflorescence with life stages of thrips. Flowering phases: (**a**) budding, (**b**) half-flowering, (**c**) full-flowering and (**d**) wilting; thrips life stages: adults (**e**), eggs (**f**, **g**) and larvae (**g**, **h**)

**Fig. 3:**
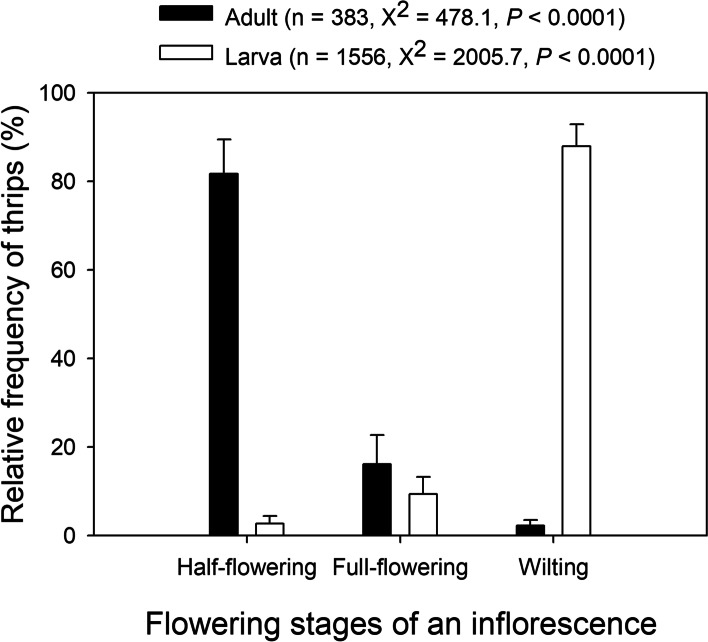
Mean relative frequencies (± SE) of thrips (adults/larvae) at different flowering stages of an inflorescence across three flowering seasons. The sample size of inflorescences was identical for the different flowering stages in each year

As shown in Figs. 2-3, specific life stages of the thrips tended to occur at specific flowering phases of *S. chamaejasme*. The adults were mainly observed in fresh flowers of the half-flowering inflorescences (Figs. 2b, e), which accounted for an average of 81.7 ± 7.8% (mean ± SE) of adult thrips surveyed in a given year, and were partly observed in flowers of the full-flowering inflorescences (Figs. 2c, e; Fig. 3). The larvae were mostly found in the wilted flowers (Figs. 2d, g, h), which accounted for 88.0 ± 4.9% (mean ± SE) of the total larvae surveyed in a given year on average. Their eggs could be observed inside floral tubes on the half- and full-flowering inflorescences (Figs. 2b, c, f, g).

### Effects of bagging on the foraging and fecundity of thrips in the host *S. chamaejasme*

**Fig. 4:**
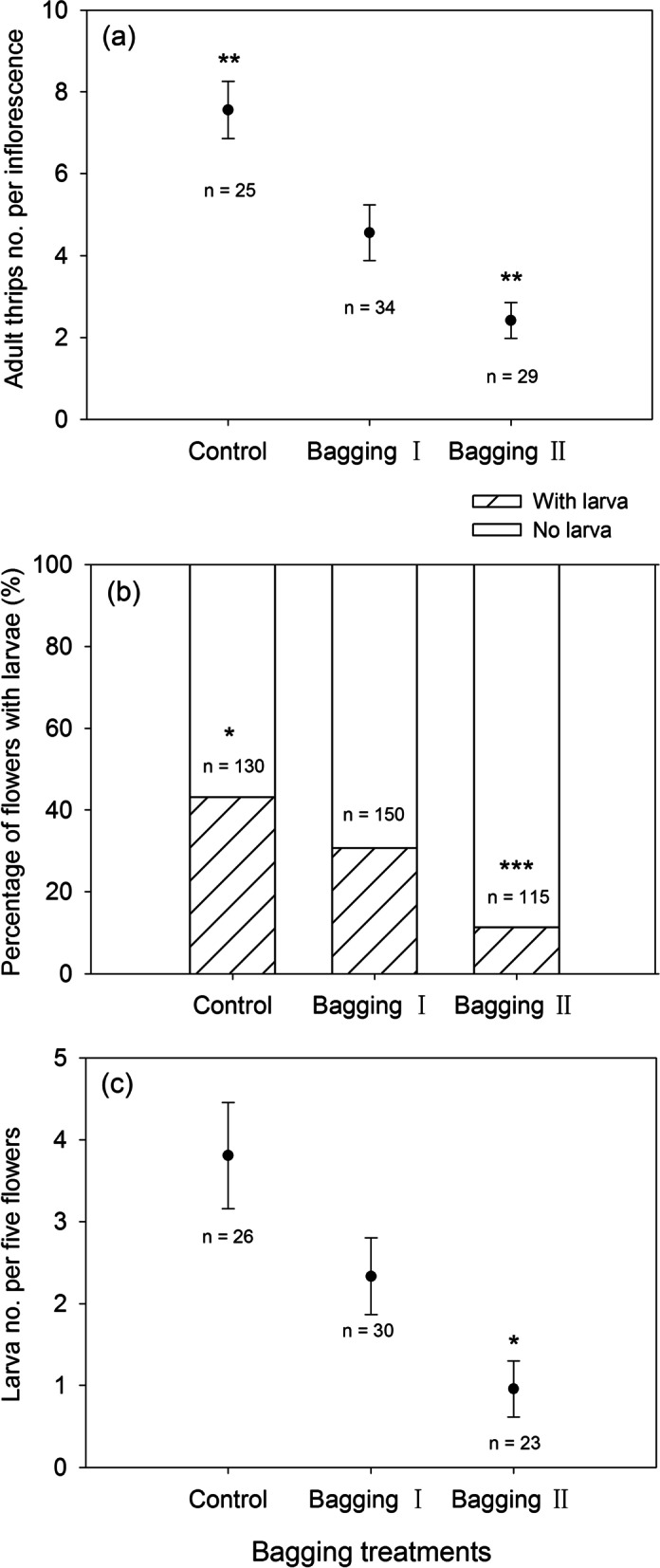
Effect of bagging treatments on thrips adults foraging flowers and their fecundity in the host *S. chamaejasme*. **a** Mean number (± SE) of thrips adults foraging flowers, (**b**) the percentage of flowers with larvae, and (**c**) mean number (± SE) of hatched larvae per five flowers. Control: open pollination; Bagging I: bagged with coarse-meshed bags; Bagging II: bagged with fine-meshed bags. Statistical significance in (**a**) and (**c**) was tested by generalized linear model (GLM) with a negative binominal error structure (log link function), and that in (**b**) by Pearson’s chi-squared test. Asterisks indicate significant difference between bagging I and either the control or bagging II (* *P* < 0.05, ** *P* < 0.01, *** *P* < 0.001)

The number of adult thrips foraging flowers of *S. chamaejasme* was significantly different among the three treatments (Fig. 4a). Under open pollination, the number of adults per inflorescence was 7.56 ± 0.70 (mean ± SE, *n* = 25), which was significantly higher than those under the two bagging treatments (*P* = 0.0058 and *P* < 0.00001, respectively). For the coarse-bagged individuals, the number of adults per inflorescence was 4.56 ± 0.68 (mean ± SE, *n* = 34), which was significantly higher than the 2.41 ± 0.44 (mean ± SE, *n* = 29) adults per fine-bagged individuals (*Z* = 2.90, *P* = 0.0037).

Under open pollination, the proportion of larva-hatching flowers (i.e., with larvae) on an inflorescence was 43.1% (*n* = 130) on average, which was significantly higher than those under the two bagging treatments (*X*^2^ = 4.63 and *X*^2^ = 30.45, *P* = 0.031 and *P* < 0.00001, respectively). The proportion was 30.7% (*n* = 150) for the coarse-bagged inflorescences and was distinctly higher than 11.3% (*n* = 115) for the fine-bagged inflorescences (*X*^2^ = 14.1, *P* = 0.00017) (Fig. 4b). Correspondingly, the number of hatched larvae per five flowers was the highest at 3.81 ± 0.65 (mean ± SE, *n* = 26) on the control inflorescences. The number was 2.33 ± 0.47 (mean ± SE, *n* = 30) for the coarse-bagged inflorescences, which did not significantly differ from the control (*Z* = 1.47, *P* = 0.14) but was significantly higher than the 0.95 ± 0.34 (mean ± SE, *n* = 23) for the fine-bagged inflorescences (*Z* = 2.29, *P* = 0.022) (Fig. 4c).

### Effect of bagging on reproduction success of *S. chamaejasme*

**Fig. 5:**
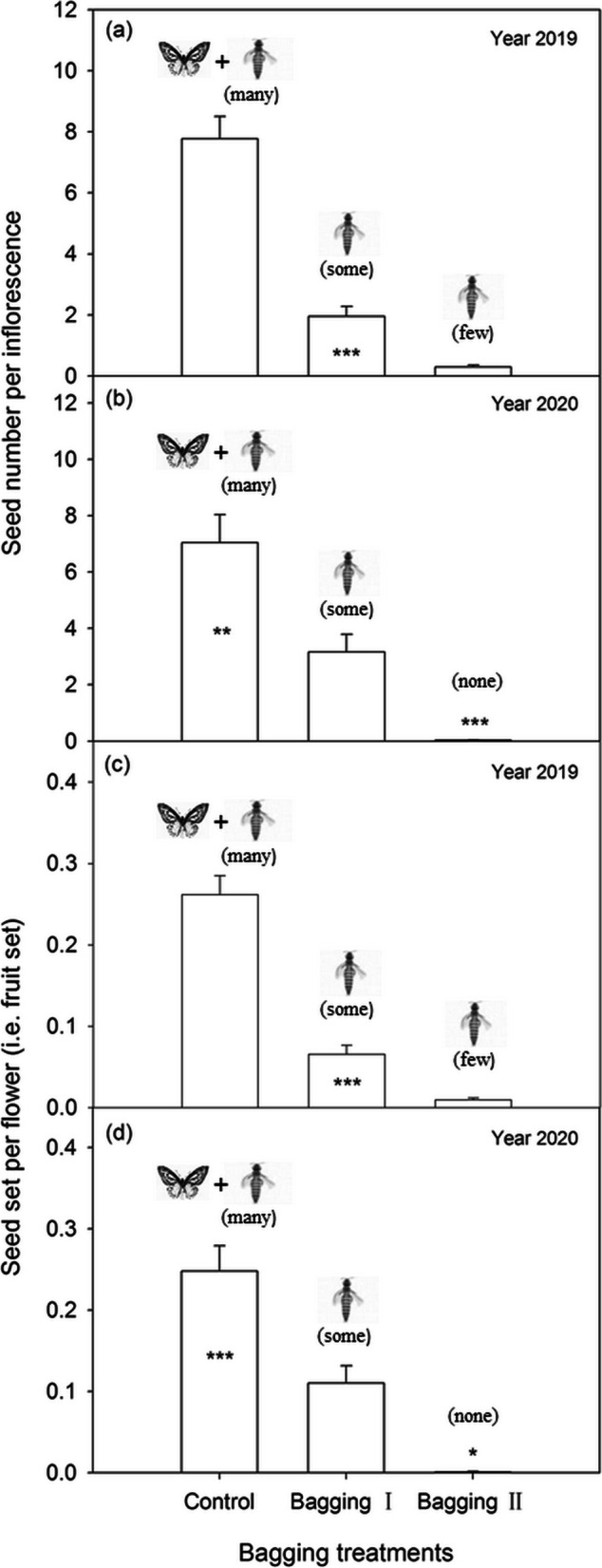
Mean seed number (± SE) per inflorescence (**a**, **b**) and seed set (± SE) per flower (**c**, **d**) of the differently-treated individuals of *S. chamaejasme*. Control: open pollination (i.e., pollinated by Lepidoptera and thrips); Bagging I: bagged with coarse-meshed bags (i.e., pollinated only by thrips); Bagging II: bagged with fine-meshed bags (i.e., almost not pollinated or by few slipped thrips). None: no pollinators. Statistical significance in (**a**) and (**b**) was tested by GLM with a negative binominal error structure (log link function), and that in (**c**) and (**d**) by GLM with a quasibinomial family (logit link function). Asterisks indicate significant difference between bagging I and either the control or bagging II (* *P* < 0.05, ** *P* < 0.01, *** *P* < 0.001)

Both seed number per inflorescence and seed set per flower (i.e., fruit set) in *S. chamaejasme* were significantly different among the three treatments (Figs. 5a-d). The open-pollinated individuals (i.e., the control) had the highest seed number per inflorescence in both flowering seasons, with values of 7.76 ± 0.73 (mean ± SE, *n* = 91) and 7.04 ± 0.99 (mean ± SE, *n* = 47), respectively (Figs. 5a, b). Additionally, the control had the highest fruit set of 0.26 ± 0.02 (mean ± SE, *n* = 91) and 0.25 ± 0.03 (mean ± SE, *n* = 47) in both seasons (Figs. 5c, d). The coarse-bagged individuals (i.e., bagging I) had a medium seed number per inflorescence and a medium fruit set in both seasons, both of which were distinctly smaller than the control in a given season (each *P* < 0.00001 except *P* = 0.0031 for seed number difference in 2020). Of the fine-bagged individuals (i.e., bagging II), both fruit set and seed number per inflorescence were the smallest, either of which was significantly lower than that of the coarse-bagged individuals in each season (each *P* < 0.00001 except *P* = 0.025 for fruit set difference in 2020).

## Discussion

### The association of *F. intonsa* brooding with flowering of *S. chamaejasme*

In brood-pollination mutualism, specific life stages of pollinating insects basically coincide with specific reproductive phases of host plants, which reflects the coevolution of two interacting partners to some extent. Generally, insect adults appear at the flowering phase of host plants as pollinators, and brooding takes place at the stage when food for their larvae is available in the hosts. In the present study, it was found that adult thrips of *F. intonsa* preferentially foraged the half-flowering inflorescences of *S. chamaejasme,* ovipositing eggs into floral tubes, and that their larvae were reared in flowers from the full-flowering to wilting phases of an inflorescence. Such recognition of specific flowering phases of host inflorescences by the adults was likely to be mediated by floral chemicals (e.g. volatiles and scents), as manifested in the mutualistic interaction between *Sambucus nigra* (elderflower) and *Thrips major* [19]. In addition, our findings indicated that a single flower of *S. chamaejasme* lasted up to 11 days on an inflorescence. Coincidently, the average time for preadult development of *F. intonsa* was 8–12 days, as documented by Ullah and Lim [23], as was the case for species such as *F. occidentalis* and *Thrips hawaiiensis* [24]. That is, the floral longevity of the host *S. chamaejasme* may exactly accommodate the insects finishing their life stages from spawning to hatching to pupation (or prepupation).

In most previously documented brood-pollination mutualisms, pollinating insects rear their offspring on seeds (or ovules) of host plants, such as classical figs-fig wasps [3], yuccas-yucca moths [5], and *Senita cacti*-*senita* moth mutualisms [12]. In the studied mutualism, however, no evidence indicated that the ovules or developing seeds of the host *S. chamaejasme* were consumed by adults and/or larvae of *F. intonsa*. It was likely that there existed a mechanism of protecting seeds against consumption in the mutualism, and the mechanism was vital to maintain mutualistic relationship between the thrips and host plants*.* Otherwise, seed predation by insects would not only impair the fitness of host plants directly but also have an adverse consequence on their own fitness. As each flower of *S. chamaejasme* produces only one ovule, damage to developing seeds (or ovules) may result in the shortened longevity of a flower, which will in return affect egg hatching and larval development in floral tubes, i.e., ultimately undermining the fecundity of the thrips. Regarding the food that host plants provide for thrips, many previous studies have documented that flower thrips can feed on pollen of a wide range of sizes [19, 25], and almost all their larvae feed on the pollen grains of host plants [2]. Therefore, it is likely that the thrips of *F. intonsa* in this mutualism were also provisioned with pollen grains of the host plants, although no direct evidence was obtained.

### Thrips pollination and its contribution to the reproduction of *S. chamaejasme*

Thrips have long been recorded as pollinators of plants from a few angiosperm families, such as Dipterocarpaceae, Winteraceae, Monimiaceae [reviewed by [22, 26]], Euphorbiaceae [17], and Caprifoliaceae [19], and also as pollinators of some gymnosperms [20, 27]. However, its pollination efficiency was not highly recognized because of its small size and cryptic behaviour until growing evidence was presented in many studies [17, 19, 28, 29]. A plant that is typically pollinated by thrips generally has a compact enclosed floral morphology together with a narrow corolla entrance, i.e., thrips pollination syndrome, which can provide shelter and brood sites for eggs and larvae of thrips [17, 30]. Consistently, the studied *S. chamaejasme* features enclosed flowers with corolla entrances (i.e., flower throat) blocked by five upper anthers, and its flowers provided brooding sites for the thrips of *F. intonsa* in this study. In addition, our findings indicated that the host plants, when foraged only by thrips, still succeeded in reproduction, and their fitness declined with the decreased occurrence of adult thrips foraging flowers. Evidently, the thrips of *F. intonsa* could serve as efficient pollen vectors and contribute to the fitness of the host *S. chamaejasme*.

Although the pollination effectiveness of the thrips was ascertained in the present study, we still underestimated its relative contribution to the reproductive fitness of host plants. The findings on pollination manipulation showed that individuals with coarse bags (i.e., bagging I), which could be foraged only by the thrips, had a much lower fitness than the open-pollinated individuals (Fig. 5). Seemingly, it was implied that the adult thrips had a low efficiency of pollination for the host plant compared to the long-tongued pollinators. Nevertheless, this was not exactly the case because the coarse bags actually not only excluded the long-tongued insects as expected but also restricted some of the thrips from entering flowers unexpectedly in this study (see Fig. 4 a). That is, the reduced fitness of the bagged individuals was greatly associated with the adverse impacts imposed by bagging on thrips foraging. Inflorescence bagging probably affected the recognition of the flowers by the thrips or physically restricted their movement to some extent [see [19, 31]]. Therefore, bagging reduced both the number of thrips entering flowers and their frequency of switching among plant individuals and consequently undermined the pollination efficiency and reproductive fitness of the plants, since the host plant is strictly self-incompatible [21].

### Maintenance of the mutualism between *S. chamaejasme* and *F. intonsa*

The trade-off in a mutualism, i.e., the costs and benefits for both interactive partners, is critical to the evolution and maintenance of mutualistic interactions [32]. In the present study, adults of *F. intonsa* pollinated the host plants of *S. chamaejasme*, and as a reward, the plants provided the thrips with breeding sites. In this mutualism, pollen consumption, as part of the benefit for the thrips, may be the main cost of host plants in obtaining pollination services. Therefore, we can postulate that retaining two whorls of 5 stamens in each flower could be an adaptive strategy of *S. chamaejasme* to maintain mutualistic relationships with the thrips and that the two whorls of stamens may be functionally divergent. The upper whorl probably functions not only as siring pollen for plant reproduction but also as a barrier at the floral throat that may protect the thrips' brood, whereas the lower whorl inside the floral tube likely serves mainly as a food source for the thrips. Taken together, the large amount of pollen produced in host flowers can not only ensure the success of plant cross-pollination but also meet the needs of thrips breeding, i.e., it can guarantee the reproductive success of both interactive partners. Certainly, further experiments are needed to confirm these hypotheses in future work.

In essence, the evolution of mutualism is based on a positive correlation in the fitness between two interactive partners [33–35], and its maintenance is conditioned upon each other’s positive contribution to their fitness. In the mutualistic system studied here, effective thrips pollination of host plants played a vital role in maintaining their mutualistic relationship. On the one hand, the number of thrips, including hatched larvae, reflected the abundance of potential pollinators of the plants, and the increased fecundity of thrips (i.e., their fitness) might correspondingly improve the fitness benefit of the host plants owing to increased flower visitation, which was partly supported by the finding in the present study. On the other hand, the growing number of thrips would result in increased pollen consumption, which would possibly lead to pollen limitation of the plants. However, pollen limitation due to thrips consumption rarely occurs in the host plants of *S. chamaejasme*, as the species produces ten anthers but only one ovule in each flower, and in such cases, its reproduction is more likely pollinator-limited than pollen-limited. In extreme cases, there exists the possibility that thrips pollination will fail, namely, that one adult of *F. intonsa* restricts its activities (e.g., foraging and ovipositing) within only one plant; in this case, the thrips will become parasitic owing to no contribution to the reproductive success of the self-incompatible plants [see [32]]. Therefore, we assume that the factors facilitating adult thrips to switch among plant individuals during their foraging and ovipositing will favour evolution of the mutualism through promoting cross-pollination of host plants. These factors probably include biotic factors, such as the foraging behaviour of *F. intonsa*, floral chemical regulation, and the flowering procedure of inflorescences, and abiotic factors, such as weather conditions (e.g., wind). In future work, relevant detailed research on the maintenance of mutualism is needed to better understand the mutualistic interaction between the host plant and thrips of *F. intonsa*.

## Conclusions

Brood pollination mutualism is a special type of plant-pollinator interaction in which insects pollinate plants as adults, and the plants provide breeding sites for the pollinators as a reward. In the present study, we manifested such a mutualistic interaction between *S. chamaejasme* and thrips of *F. intonsa*. The adult insects preferentially foraged the half-flowering inflorescences of the plants, pollinating host flowers and ovipositing in floral tubes. Floral longevity might exactly allow the thrips to finish their life cycle from spawning to prepupation. The exclusion of adult thrips from foraging flowers led to a significant decrease in the fitness of host plants and a corresponding reduction in thrips fecundity (i.e., larva no. per floral tube). In the studied interaction, there probably existed a mechanism of protecting seeds (or ovules) of host plants from consumption by the thrips, which was vital to maintain their mutualistic relationship. In conclusion, the thrips of *F. intonsa* and the host *S. chamaejasme* interact mutualistically to contribute to each other’s fitness in that the thrips pollinate host plants as adults, and as a reward, the plants provide the insects with brooding sites and food, indicating coevolution of the thrips' life cycle and the reproductive traits (e.g., floral longevity and morphology) of *S. chamaejasme*.

## Materials and methods

### Study site and materials

*S. chamaejasme* is distributed geographically from southern Russia, across Mongolia, over northern and southwestern China (including the Tibetan Plateau), and southwards to the western Himalayas [21, 36]. The study site, which belongs to the protected area of the Qilian Mountains national nature reserve, is located in an alpine meadow to the north of the Tianzhu field station of Gansu Agricultural University (37°12′50″N, 102°47′33″E; 3100 m a.s.l.), Tianzhu county, Gansu Province, China. The studied population is white-flowered, and each individual produces 15–20 capitate inflorescences (Fig. 1a). Each capitate head has an average of 20–30 flowers, and each flower produces only one ovule and two whorls of 5 stamens (i.e., 10 stamens in total), with the upper whorl at the flower throat and the lower whorl in the inner middle wall of the floral tube (virtually a calyx tube). A voucher specimen of this material, which was collected in 2019 and identified by Qiang-En Fang, has been deposited in the herbarium of the Northwest Institute of Eco-Environment and Resources of Chinese Academy of Sciences (LZD, 0050602).

### Experimental approach

In this study, the phenology of an inflorescence was divided into four phases according to the number of open flowers: budding, half-flowering (i.e., marginal flowers on the head have opened), full-flowering (i.e., all flowers on the head have opened) and wilting (Fig. 2). To determine the association of the life cycle of the thrips with the flowering phenology of *S. chamaejasme*, we first labelled 50 budding inflorescences at the beginning of the flowering season in 2017 and measured the time that each flowering phase lasted by recording the dates when the first flower, half of the flowers and all of the flowers opened and the date when the last flower withered on each inflorescence. Second, we measured the occurrences of adult thrips and larvae in the different flowering phases. In three consecutive flowering seasons (i.e., year 2017–2019), we randomly sampled inflorescences that were at different flowering stages (the sample size was identical for each flowering phase); then, we counted the number of adult thrips on each sampled inflorescence by shaking them into a 100-mesh (≤ 0.2 mm) bag, and counted the number of larvae in 5 randomly selected flowers (i.e., florets) of each inflorescence. Finally, some inflorescences at different flowering phases were collected for the observation of thrips eggs by dissecting the inner tissue of floral tubes in the lab, followed by collecting images under a dissecting microscope (Zeiss stereomicroscope with AxioVision SE64 Rel 4.8 system, Carl Zeiss Microscopy GmbH, Gottingen, Germany).

Apart from observation of the thrips, we surveyed other potential pollinators (e.g., lepidopteran insects) of *S. chamaejasme.* We chose three sunny days (each time from 10:00 to 17:00) in the full-flowering period and recorded the frequency of pollinators of each Lepidoptera species that occurred and foraged flowers in a defined area (5 × 5 m^2^) of the population. To determine the relative contribution of thrips and lepidopteran pollinators to plant pollination, we conducted a pollination manipulation experiment with pollinator exclusion treatments in two consecutive years (i.e., year 2019 and 2020). In the experiment, two types of bags were employed, i.e., coarse-meshed (5–6 mm) and fine-meshed (0.15–0.2 mm) bags. Over 300 budding inflorescences, which were randomly distributed in the population and flowered synchronously, were labelled with plastic tags. The labelled inflorescences were randomly assigned to one of three treatments: the control (open pollination); bagging I (i.e., bagging with coarse-meshed bags), which could exclude lepidopteran pollinators and leave thrips for foraging, to determine sole contribution of the thrips to pollination; and bagging II (i.e., bagging with fine-meshed bags), which could exclude both lepidopteran insects and thrips, with the purpose of testing whether the plant could reproduce without insect pollination (e.g., apomixes). We bagged each inflorescence at the budding stage (i.e., before anthesis), preceded by shaking off the thrips that had infested the inflorescence previously. To check the effectiveness of bagging treatments for pollinator manipulation, we sampled a portion of labelled inflorescences from each treatment (each sample size > 20) and counted the number of adult thrips on the inflorescences at the half-flowering stage as described above; these sampled inflorescences were not used for fitness measurements of the plants. Additionally, to determine the impact of bagging on the fecundity of thrips, we sampled another portion of inflorescences from each treatment (each sample size > 20) and counted the number of larvae hatched in floral tubes by randomly selecting 5 flowers on each inflorescence at the wilting stage and then calculated the percentage of flowers with larvae out of the total surveyed flowers for each treatment. Bags were not removed until the seeds were harvested. Approximately 7–10 days after flower wilting, each tagged inflorescence was collected, followed by counting the number of filled seeds on each inflorescence.

In the pollinator manipulation experiment during the first year, we found that fine-meshed bags (i.e., bagging II) did not completely exclude thrips from foraging flowers, very few thrips entered the bags along the stem of an inflorescence via bag mouths, and the bagged plants set seeds in consequence. That is, we could not affirm that the plants do not reproduce without insect pollination. Therefore, we repeated the experiments on pollinator manipulation and its effect on the reproductive fitness of host plants in the next year, and we closed the bag mouths more tightly than in the previous experiment. In both flowering seasons, we obtained similar data from the pollinator manipulation (bagging) except that bagging II excluded almost all thrips from foraging, as expected, in the second year. As such, to keep the results on adult thrips’ foraging and their fecundity (i.e., larva no.) on the inflorescences comparable, namely, by using related data obtained in a given season, we presented 1 year (i.e., the first season) of data on the number of adult thrips foraging on inflorescences under different treatments and 2 years of data on the effect of bagging on the reproductive fitness of the plants in the results of the present study.

### Data analysis

We calculated both the mean relative frequency of adult thrips foraging inflorescences and the mean relative number of larvae hatched in inflorescences at each flowering phase in three consecutive years. Then, we pooled the data of the 3 years and used Pearson’s chi-squared test to determine the difference in the frequency distribution of thrips (adults/larvae) among different flowering phases. We also used Pearson’s chi-squared test to determine the difference in the percentage of larva-hatching flowers on an inflorescence among treatments. For count data of pollination manipulation, that is, the number of adult thrips foraging flowers, the number of hatched larvae in flowers, and the seed number per inflorescence (i.e., the sum of the seeds of all flowers), we employed a generalized linear model (GLM) with a negative binominal error structure (log link function) to test the significance of differences among treatments. Because each flower has only one ovule, we used a GLM with a quasibinomial family (logit link function) to determine the difference in seed set per flower among treatments, with the treatments as fixed effects. For all data analyses, R version 3.5.0 was used, and no transformations were performed.

## Supplementary Information

**Additional file 1.**

**Additional file 2.**

## Data Availability

All data generated or analyzed during this study are included in this published article and its supplementary information files.

## Abbreviations

GLM: Generalized linear regression
SE: Standard error
